# Supplementary material for: The voltage sensing phosphatase (VSP) localizes to the apical membrane of kidney tubule epithelial cells
Source: PLoS One. 2019 Apr 9;14(4):e0209056. doi: 10.1371/journal.pone.0209056 (PMC6456211; doi:10.1371/journal.pone.0209056)
Supplement: S1 Table — (DOCX) [file pone.0209056.s001.docx]

Supplementary Table 1 | Voltage dependence of activity

| VSP | fTAPP | | | fPLC | | gPLC | |
| --- | --- | --- | --- | --- | --- | --- | --- |
|  | n | V_1/2_ up | V_1/2_ down | n | V_1/2_ down | n | V_1/2_ up |
| Xl-VSP1 | 10 | 55 ± 2 | 97 ± 3 | 11 | 98 ± 5 | 11 | 77 ± 3 |
| Xl-VSP2 | 11 | 92 ± 8 | n/a | 11 | 83 ± 6 | 9 | 118 ± 3 |
