# Supplementary figures and images for: The voltage sensing phosphatase (VSP) localizes to the apical membrane of kidney tubule epithelial cells
Source: PLoS One. 2019 Apr 9;14(4):e0209056. doi: 10.1371/journal.pone.0209056 (PMC6456211; doi:10.1371/journal.pone.0209056)

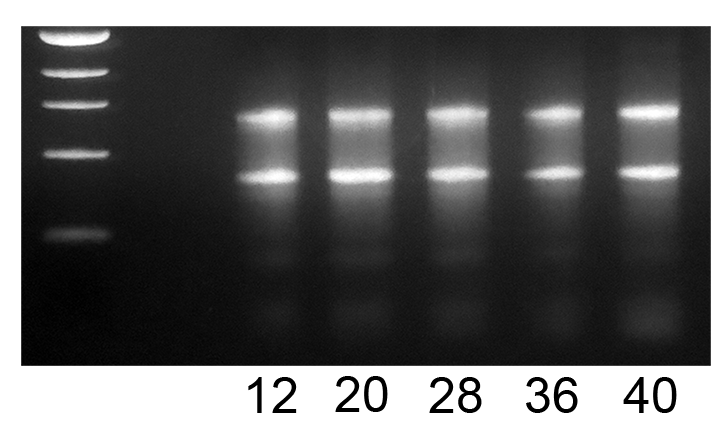

Supplement: S1 Fig — RNA concentrations were determined by Nanodrop spectrophotometry and 1 μg of RNA was run on a 2% agarose gel stained with ethidium bromide. The prominent bands seen here are the 28S and 18S ribosomal RNAs, indicating that the RNAs are not degraded and that approximately equal total RNA was used for subsequent sqRT-PCR analysis. (TIF) [file pone.0209056.s002.tif]

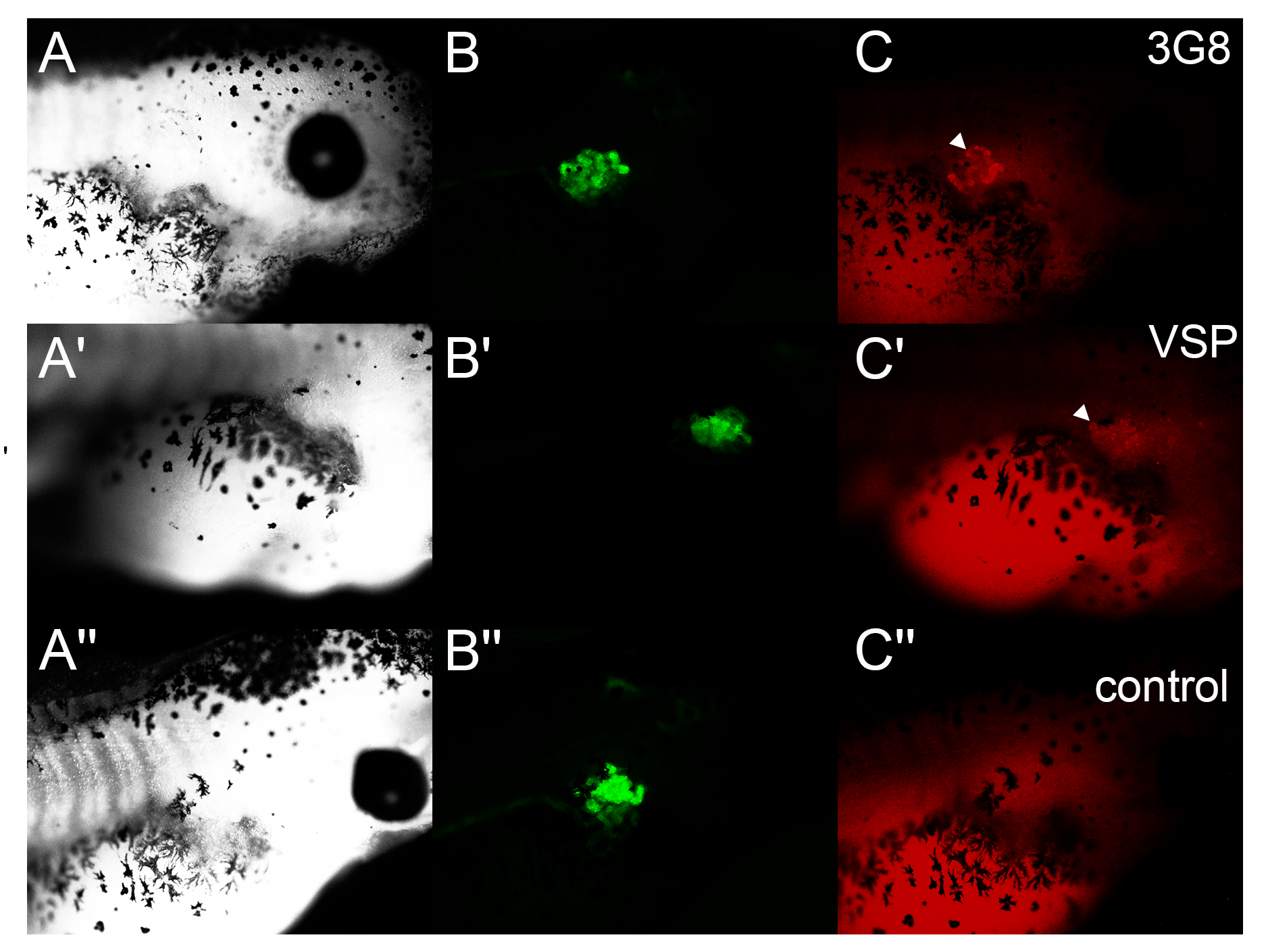

Supplement: S2 Fig — Embryos stained with 3G8 antibody (A-C), anti-VSP (A'-C') or secondary antibody alone control (A''-C''). The Pax8:GFP embryos show strong GFP expression in the proximal pronephros (B-B'') as confirmed by staining with the proximal pronephros marker 3G8 antibody (C). Xl-VSP staining of tubules is clearly visible (C') and co-localizes with the GFP, indicating Xl-VSP in the proximal pronephros. Positive staining is marked with arrowheads, whereas no signal above autofluorescence was seen in control animals stained with secondary antibody alone (C''). (TIF) [file pone.0209056.s003.tif]

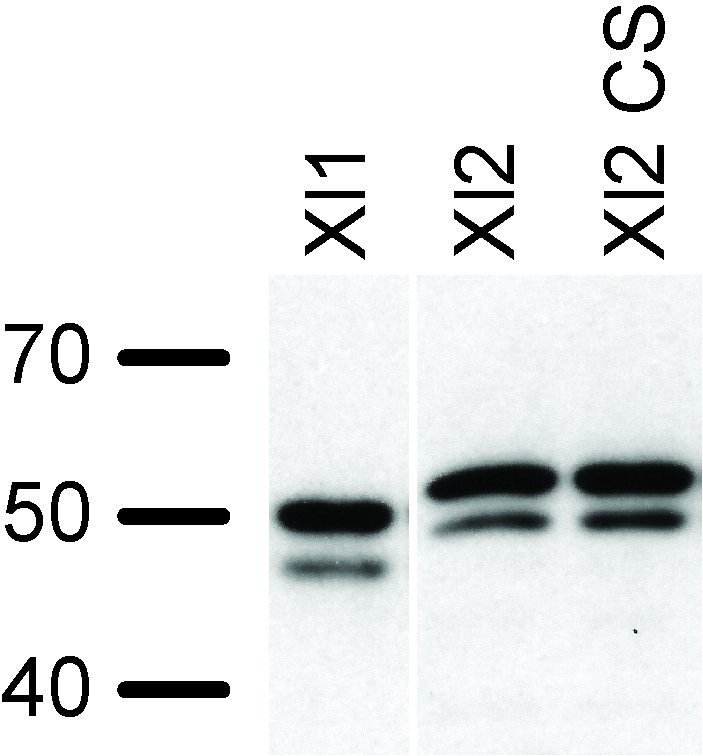

Supplement: S3 Fig — Individual X. laevis oocytes used for the activity assay in Fig 6 were probed for protein expression by Western blot with anti-VSP N432/21. Oocytes were injected with cRNA for wild type Xl-VSP1 (Xl1), wild type Xl-VSP2 (Xl2) and catalytically dead Xl-VSP2 C301S (Xl1-CS). (TIF) [file pone.0209056.s004.tif]
